# Is Ultra-Processed Food Intake Associated with a Higher Risk of Glaucoma? A Prospective Cohort Study including 19,255 Participants from the SUN Project

**DOI:** 10.3390/nu16071053

**Published:** 2024-04-04

**Authors:** José Francisco López-Gil, Alejandro Fernandez-Montero, Maira Bes-Rastrollo, Laura Moreno-Galarraga, Stefanos N. Kales, Miguel Ángel Martínez-González, Javier Moreno-Montañés

**Affiliations:** 1 One Health Research Group, Universidad de Las Américas, Quito 170124, Ecuador; josefranciscolopezgil@gmail.com; 2 Department of Environmental Health, T.H. Chan School of Public Health, Harvard University, Boston, MA 02138, USA; skales@hsph.harvard.edu; 3 Department of Occupational Medicine, University of Navarra, 31008 Pamplona, Spain; 4 Department of Preventive Medicine and Public Health, School of Medicine, University of Navarra, 31008 Pamplona, Spain; mbes@unav.es (M.B.-R.); mamartinez@unav.es (M.Á.M.-G.); 5 Instituto de Investigación Sanitaria de Navarra (IdiSNA), 31008 Pamplona, Spain; lauramoreno11@yahoo.es (L.M.-G.); jmoreno@unav.es (J.M.-M.); 6 CIBER Fisiopatología de la Obesidad y Nutrición (CIBER Obn), Instituto de Salud Carlos III, 28029 Madrid, Spain; 7 Department of Pediatrics, Complejo Hospitalario de Navarra, Servicio Navarro de Salud, 31008 Pamplona, Spain; 8 Department of Nutrition, T.H. Chan School of Public Health, Harvard University, Boston, MA 02138, USA; 9 Department of Ophthalmology, Clínica Universidad de Navarra, 31008 Pamplona, Spain

**Keywords:** NOVA food classification system, eye diseases, glaucoma, eating healthy, Mediterranean cohort

## Abstract

Objective: The aim of this study was to examine the relationship of ultra-processed food (UPF) intake with the incidence of glaucoma in a large sample of Spanish university graduates followed prospectively. Methods: Prospective cohort study using data from the SUN Project. A final sample of 19,225 participants (60.1% women) was included in this study, with a mean age of 38.2 years (standard deviation (SD) = 12.4). Participants were followed-up for a mean time of 12.9 years (SD = 5.4). Dietary intake was measured using a 136-item semiquantitative food-frequency questionnaire. UPFs were defined based on the NOVA classification system. Glaucoma diagnosis was determined by asking the participants if they had ever been diagnosed with glaucoma by an ophthalmologist. This self-reported diagnosis of glaucoma has been previously validated. Results: After adjusting for several covariates, participants with the highest UPF consumption were at higher risk of glaucoma (hazard ratio (HR) = 1.83; 95% confidence interval (CI) 1.06 to 3.17) when compared to participants in the lowest category of UPF consumption. Regarding subgroup analyses, a significant multiplicative interaction was found for age (*p* = 0.004) and omega 3:6 ratio (*p* = 0.040). However, an association between UPF consumption and glaucoma was only found in older participants (aged ≥ 55 years), in men, in the most physically active group, in the group of non- or former smokers, in those with a lower omega 3:6 ratio, and in those with a lower energy intake. Regarding the contribution of each type of UPF group, UPF coming from sweets showed a significant risky effect (HR = 1.51; CI 95% 1.07 to 2.12). Conclusions: This prospective cohort study shows that participants with a greater UPF consumption have a higher risk of developing glaucoma when compared to participants with a lower consumption. Our findings emphasize the relevance of monitoring and limiting the consumption of UPFs as a means of preventing glaucoma incidence.

## 1. Introduction

Glaucoma is a significant cause of permanent blindness worldwide [1], characterized by a group of eye conditions that result in the progressive loss of retinal ganglion cells [1]. These cells are located within the retina and are responsible for connecting to the optic nerve [2]. Glaucoma is a widespread condition affecting over 70 million individuals globally. Of these, approximately 10% are completely blind in both eyes, making it the primary reason for permanent blindness around the world [3]. The number of people affected by glaucoma is projected to increase it was estimated that a total of 60.5 million people were affected by glaucoma in 2010, increasing to 79.6 million in 2020 [3].

Research in the scientific community has identified several factors that increase the risk of glaucoma, such as elevated intraocular pressure, advanced age, non-Caucasian race, and family history of the condition [1]. However, there is growing recognition of the impact that modifiable environmental factors, such as nutrition, exercise, and lifestyle can have on the development of glaucoma [4]. As a result, the use of alternative and complementary medicine in the treatment of glaucoma has become of great attention to both patients and ophthalmologists [5]. Despite the available evidence, additional investigation is warranted to fully comprehend the therapeutic potential of these treatments through both laboratory research and well-designed clinical studies [5].

Regarding nutrition, the concept that ultra-processed foods (UPFs) are an unhealthy component of people’s diets is gaining widespread recognition in the field of nutrition research and official reports [6,7,8,9]. UPF has been defined as a type of food that is made up of industrial formulations primarily consisting of food-derived substances, additives, and other artificial ingredients [10]. These foods are designed to be convenient, long-lasting, and very tasty, but they often contain high levels of salt, sugar, and fat, while providing little nutritional value, such as snacks, sweetened beverages, frozen meals, or fast food [11]. There is increasing awareness of how UPFs can negatively impact the quality of a person’s diet and increase the likelihood of health issues [12,13]. Worldwide, from 1990 to 2010, there was a notable rise in the consumption of less nutritious food products, exhibiting variation across regions and nations [14]. UPFs have become ubiquitous in global diets, representing anywhere from 20% to over 60% of total energy intake, the extent of which varies by country and age group [8]. In Spain, one previous study, including four representative Spanish cohorts, reported a total increase of 10.8% in UPF consumption between 1991 and 2008, which is in line with similar studies conducted in this same country [15,16]. Despite this, UPFs have received limited attention in efforts to improve overall health [11]. To address this gap, it is crucial to provide evidence that links UPF consumption to health outcomes.

Although there is limited evidence linking diet to glaucoma, previous research has suggested connections between nutrition and glaucoma risk [17,18,19,20]. For example, a greater consumption of carbohydrates has been related to an increased risk of developing glaucoma [17]. Additionally, the dietary ratio of omega 3:6 fatty acid intake may impact the balance of intraocular pressure, which is the most significant modifiable risk factor for glaucoma [18]. Other factors that may increase the risk of glaucoma involve low selenium and iron intake [19]. On the other hand, certain nutrients, such as nitric oxide found in dark green leafy vegetables and vitamins A, C, and E, may have a protective effect against glaucoma [20]. However, the association between UPF consumption and glaucoma risk is still unclear. Due to the limited strength of evidence linking diet to glaucoma, further research is necessary to make these findings applicable to clinical practice [19]. Therefore, the aim of the current study was to examine the relationship of UPF consumption with the incidence of glaucoma in a large sample of Spanish university graduates followed prospectively.

## 2. Methods

### 2.1. Study Design and Population

The *Seguimiento Universidad de Navarra* (SUN) Project is a large prospective cohort study that focuses on Spanish university graduates in a Mediterranean setting. Its purpose is to identify the lifestyle and dietary variables that contribute to various diseases, such as cardiovascular disease, mental illness, and cancer. The study was initiated in 1999 and remains an ongoing open cohort. Participants are followed up by biennial questionnaires that can be completed through the mail or online. Information on the SUN cohort profile can be accessed at www.medpreventiva.es/xZd6Hh. 

The first questionnaire (referred to as Q0 or baseline questionnaire) contains information on the participants’ sociodemographic characteristics, physical measurements, diet, eating habits, lifestyle practices, and medical history. Follow-up questionnaires, which are sent every two years (Q2–Q20), assess changes in lifestyle, diet, and medical information and monitor the incidence of new diseases. After 10 years of follow-up, the Q10 questionnaire was used to provide updated information on the participants’ behaviors and lifestyles. Participants were given an understanding of the information and SUN Project methods before completing Q0, implying their informed consent. Participants were requested to give their explicit permission prior to accessing their medical records or incorporating them into validation studies and were informed of their right to decline participation or withdraw their consent at any time, as per the Declaration of Helsinki. The SUN Project was approved by the University of Navarra Institutional Review Board (091/2008), approved on 18 April 2011.

**Figure 1:**
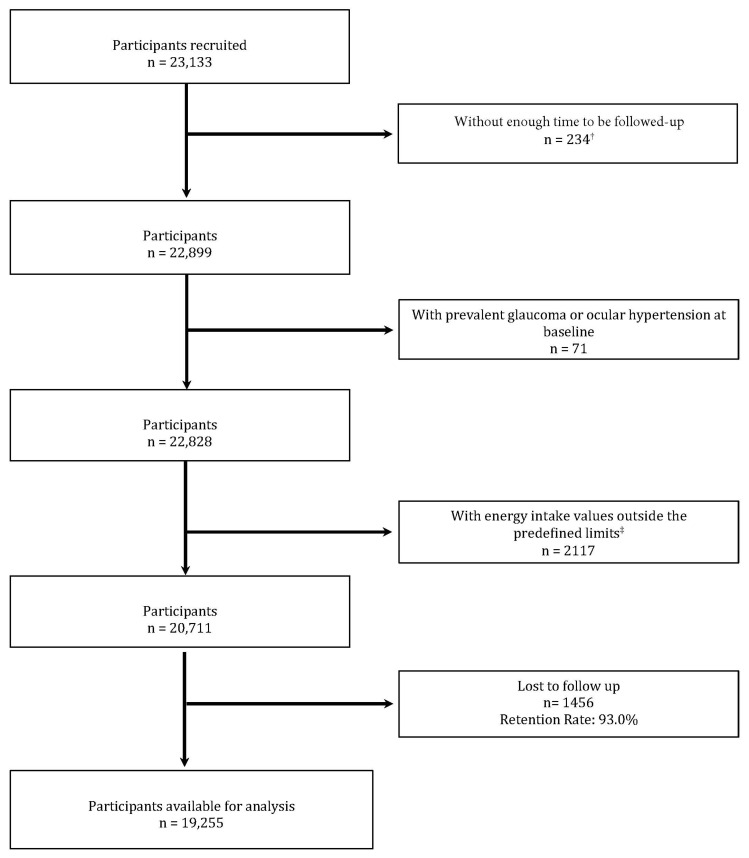
Flow diagram illustrating the participation process in the SUN Project. ^†^ These are participants for whom baseline information is available but who have not yet received follow-up information for the first two years. ^‡^ Values outside of predefined limits according to Willett [26]: <800 kcal/day for men and <500 kcal/d for women; >4000 kcal/day for men and >3500 kcal/day for women.

As of September 2019, 22,899 individuals had joined the SUN Project. However, those with glaucoma at the start of the study and individuals who met other exclusion criteria were excluded from the analysis, which resulted in 19,225 participants being included in the study (Figure 1).

### 2.2. Glaucoma Incidence (Dependent Variable)

The diagnosis of glaucoma was evaluated at the beginning of the SUN Project (Q0) and every two years during the follow-up. Participants who had already been diagnosed with glaucoma at baseline were excluded from the analysis. The participants were asked if they had ever been diagnosed with glaucoma by an ophthalmologist as follows: “Have you ever been diagnosed with glaucoma by a health care professional?”. Additionally, the date of diagnosis was also recorded. 

A subsample of 150 participants was clinically evaluated by an ophthalmologist to validate the self-reported diagnosis of glaucoma. Glaucoma was defined as per the recommendations of the European Glaucoma Society [21], as a condition characterized by damage to the edge of the optic nerve and loss of retinal nerve fibers, resulting in a visual field impairment. The validation results showed high agreement between self-reported and clinical diagnoses (kappa (*κ*) = 0.85; 95% CI 0.83 to 0.87) and high specificity (0.99) and sensitivity (0.83). All validated diagnoses were open-angle glaucoma [17].

### 2.3. UPF Consumption (Independent Variable)

Dietary intake was measured at the beginning of the study using a 136-item semiquantitative food-frequency questionnaire (FFQ). This questionnaire has been validated previously in Spain and was used to assess the participant’s typical food consumption [22,23]. The questionnaire was repeated after 10 years to account for any changes in the participant’s diet. To reduce the impact of changes in diet over time, both sets of dietary data from the two FFQs were used in the repeated measure analyses. The FFQ recorded the standard portion size of each food item in Spain and asked about the frequency of consumption, which was divided into nine categories varying from never/almost never to more than six servings daily. The daily food consumption of the participant was determined by multiplying the amount of each food item consumed with the frequency of how often it was consumed.

UPFs were defined based on the NOVA classification system [10], which categorizes foods into four groups based on the level and purpose of industrial processing. The four categories are (1) unprocessed or minimally processed foods, (2) processed culinary ingredients, (3) processed foods, and (4) UPF and drink products [24,25]. Further information about the NOVA classification system in the SUN project can be found in Table 1.

The amount of UPF consumed by each person (in grams daily) was computed by adding up the amount of each UPF item listed in the Table 1. The total consumption of UPFs was corrected for the overall energy intake for each person, separately for men and women, using a statistical method called the residual method [26]. Participants were divided into four groups according to the daily number of servings of UPFs consumed, with the first group consuming one serving or less, the second group consuming more than one to three servings, the third group more than three to four servings, and the fourth group more than four servings. In addition, to evaluate the contribution of each different type of UPFs with the risk of glaucoma, we performed a further classification (Supplementary Table S1).

### 2.4. Covariates

The study gathered information on various factors that could influence the results, acting as confounding factors. These factors included sociodemographic information such as the participant’s age, sex, and education level; lifestyle and dietary variables such as total energy intake, adherence to the Mediterranean diet, physical activity, smoking, ethanol intake, caffeine intake, dietary omega 3:6 ratio, and special diets; anthropometric measures such as body mass index; and the participant’s medical history, including any previous diagnoses of diseases such as cardiovascular disease, cancer, hypertension, or diabetes.

### 2.5. Statistical Analysis

The study used Cox regression models to examine repeated measures of the relationship between UPF consumption and glaucoma risk. The study compared the incidence of glaucoma between the lowest category of UPF servings consumption, selected as the reference group, and the other three groups of higher UPF consumption. The results were measured by hazard ratios (HRs) with their 95% confidence intervals (CI). The models were adjusted for various confounding factors, such as demographics (sex, age, educational level), lifestyle and dietary (physical activity, total energy intake, adherence to the Mediterranean diet, smoking, ethanol intake, caffeine intake, dietary omega 3:6 intake ratio and special diets), anthropometric (body mass index), and self-reported medical history (cancer, diabetes, hypertension, and cardiovascular disease). All models were stratified by decades of age and recruitment period. Furthermore, subgroup analyses and examinations for mutual influence (i.e., interaction) were also performed by sex (men and women), age (<55 years and ≥55 years), physical activity (<50th percentile and ≥50th percentile), tobacco use (never/former and smokers), omega 3:6 ratio (<50th percentile and ≥50th percentile), and energy intake (<50th percentile and ≥50th percentile). Moreover, further analyses were conducted to evaluate the impact of different types of UPFs (i.e, “sweets”, “sausages”, “beverages”, “fried foods”, “fast food”, and “dairy products”) on the risk of glaucoma, stratifying the consumption of each specific type of UPFs into quartiles. These analyses were further adjusted for the number of servings of the rest of the UPF group (i.e., if “sweets” were evaluated, analyses were additionally adjusted for the sum of “sausages”, “beverages”, “fried foods”, “fast food”, and “dairy products”). Statistical significance was considered if the *p* value was less than 0.05. All analyses were conducted with STATA 17.0 (Stata, College Station, TX, USA) software for Windows.

## 3. Results

In this study, among the participants, 60.1% were female, with an average age of 38.2 years (standard deviation (SD) = 12.4). Over a mean follow-up period of 12.9 years (SD = 5.4), 230 new cases of glaucoma were documented, with a total of 176,963 person-years studied. Descriptive data of the study participants (according to their UPF consumption) are shown in Table 2. Participants with the highest consumption of UPF were younger, drank more ethanol, and had a higher caffeine and energy intake.

Table 3 shows the risk of glaucoma according to UPF consumption by servings/day. The full adjusted model (including sociodemographic information, lifestyle and dietary variables, medical history) showed that participants with a consumption of more than four servings of UPF per day were at higher risk of glaucoma (HR = 1.84; 95% CI 1.06 to 3.21) compared to participants with a consumption of up to one serving of UPF per day (*p* for trend = 0.005).

**Figure 2:**
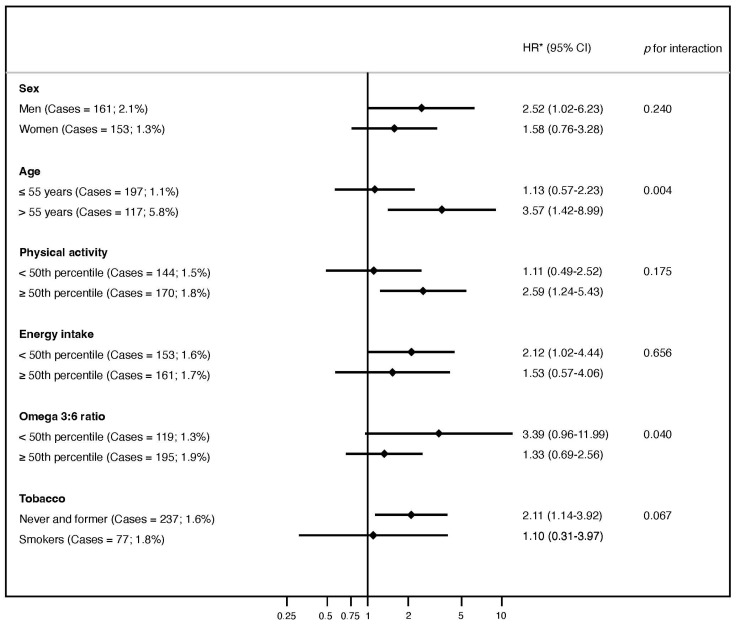
Subgroup analyses of the association between ultra-processed food intake (>4 servings/day versus up to 1 serving/day) and the risk of glaucoma according to sex, age, physical activity, tobacco use, omega 3:6 ratio, and energy intake. Data are expressed as the hazard ratio of the highest ultra-processed food (fourth quartile). The lowest quartile (first quartile) was selected as the reference group. CI, confidence interval; HR, hazard ratio; UPF, ultra-processed food. * Adjusted for sex, age, educational level, physical activity, total energy intake, adherence to the Mediterranean diet, smoking, ethanol intake, caffeine intake, dietary omega 3:6 intake ratio, special diets, body mass index, cancer, diabetes, hypertension, and cardiovascular disease.

In Figure 2, subgroup analyses illustrate the association between UPF consumption (>4 servings/day versus up to 1 serving/day) and the risk of glaucoma. After stratification by age, sex, physical activity, tobacco use, omega 3:6 ratio, and energy intake, a significant multiplicative interaction was found for age (*p* = 0.004) and omega 3:6 ratio (*p* = 0.040). However, an association between UPF consumption and glaucoma was only found in older participants (aged ≥ 55 years), in men, in the most physically active group, in the group of non- or former smokers, in those with a lower omega 3:6 ratio, and in those with a lower energy intake. 

**Figure 3:**
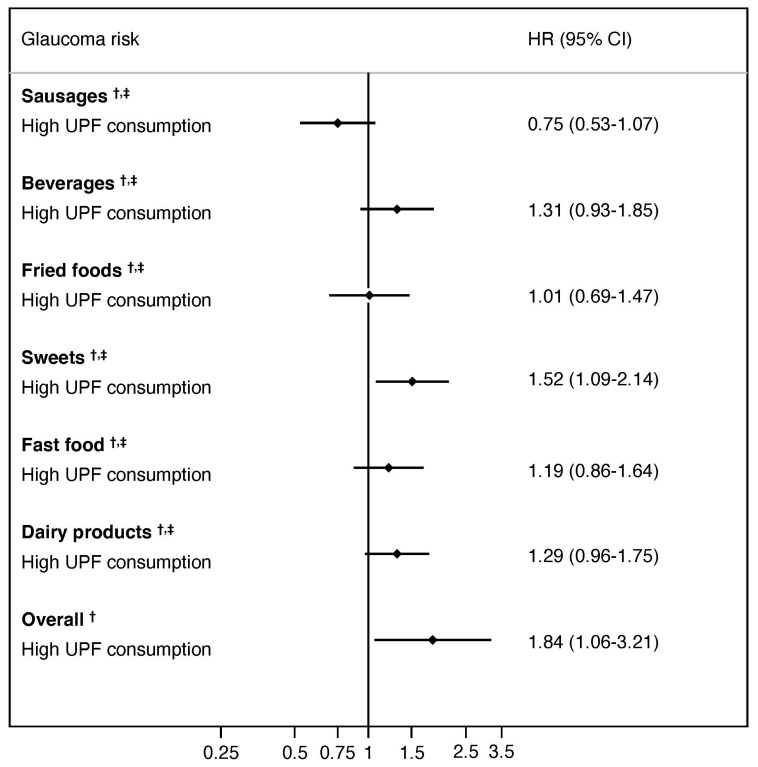
Contribution of each type of ultra-processed food group to the association between ultra-processed food intake (fourth quartile versus first quartile of UPF consumption) and the risk of glaucoma. CI, confidence interval; HR, hazard ratio; UPF, ultra-processed food. ^†^ Adjusted for sex, age, educational level, physical activity, total energy intake, adherence to the Mediterranean diet, smoking, ethanol intake, caffeine intake, dietary omega 3:6 intake ratio, special diets, body mass index, cancer, diabetes, hypertension, and cardiovascular disease. ^‡^ Further adjusted for the number of servings of the rest of the UPF group (i.e., if “sweets” were evaluated, analyses were additionally adjusted for the sum of “sausages”, “beverages”, “fried foods”, “fast food”, and “dairy products”).

Figure 3 shows the risk of glaucoma for each UPF group (fourth quartile versus first quartile of UPF consumption). When each UPF group was analyzed individually (i.e., “sausages”, “beverages”, “fried foods”, “sweets”, “fast food”, “dairy products”), apart from the global effect of all groups together, only UPF from group of sweets showed a significant effect (HR = 1.52; 95% CI, 1.08 to 2.14).

## 4. Discussion

To our knowledge, this study is the first to analyze the association between UPF consumption and glaucoma risk. Overall, we identified that participants with the highest UPF consumption were at higher risk of developing glaucoma compared to those with the lowest UPF consumption. The results remained significant even after considering several relevant covariates. Furthermore, when analyzed independently, UPF from sweets showed a significant glaucoma risk. Although the mechanisms by which food/nutrients may increase the risk of glaucoma are not fully understood [5], we propose some possible hypotheses that could justify the results found.

A possible explanation for the findings could lie in the increase in blood glucose levels caused by higher UPF consumption. When analyzing the results by type of UPF, only the consumption of sweets was found to be statistically significant. This is in line with previous research by Fardet et al. [27] stating that the more a food is processed, the lower its nutrient density and the greater its glycemic impact. Furthermore, one previous study in Australia also reported that UPF contributes to excessive sugar intake [28]. High blood sugar levels, along with oxidative stress and limited cell division in many eye tissues, can lead to the formation and accumulation of advanced glycation end products, which can cause damage to eye tissues [29]. UPF consumption is also a major source and contributor of dietary advanced-glycation end products [30]. As a result, eye tissues become susceptible to damage caused by glycation [29]. Although this may be an important explanation for the effect, it should be borne in mind that the risk effect of all UPF foods is greater than that of the group with the highest consumption of sweets, so there may be other biological mechanisms involved.

On the other hand, the role of oxidative stress and inflammation cannot be ruled out as a possible explanation for these results. The retina is exposed to reactive oxygen species (ROS) due to intense mitochondrial activity, and oxidative stress is considered a significant risk factor for glaucoma [31]. A persistent inflammation can result from an imbalance between ROS production and elimination by defensive mechanisms [32]. As previously mentioned, consuming a high amount of UPF may lead to weight gain, endothelial dysfunction, increased blood glucose levels, oxidative stress, and inflammation (among other health problems) [33]. The link between UPF consumption and low-grade inflammation is not fully understood and is only partially attributed to the high proinflammatory properties of these types of foods [34]. Additionally, UPF consumption is likely related to the resting metabolic rate, which is mediated through variations in the production of high-sensitivity C-reactive protein (hs-CRP), monocyte chemoattractant protein-1 (MCP-1), plasminogen activator inhibitor-1 (PAI-1), and interleukin-1beta (IL-1β) [35]. UPF consumption has also been linked to an increase in interleukin-6 (IL-6) levels [36], which has been linked to the survival and degeneration of retinal ganglion cells and the development of glaucoma [37]. The characteristics of retinal ganglion cell axonopathy, such as reduced axon transport and degeneration of axon structure, probably result from separate processes, with IL-6 playing a role in the specific mechanism causing degeneration of axon structure [37].

On the other hand, UFPs typically contain a wide variety of additives and artificial substances, which are added to improve flavor, texture, appearance, and shelf life (among other reasons) [38]. Some examples of common additives found in UPFs are artificial sweeteners, emulsifiers and stabilizers, flavor enhancers, preservatives, colorings, and texturizers [10]. One of the mechanisms by which UPFs may contribute to these conditions is through the promotion of inflammation [8]. Artificial sweeteners, thickeners, emulsifiers, and preservatives may have indirect and direct effects on immune cells, contributing to metabolic dysregulation [39]. We hypothesized that increased consumption of these types of foods, rich in these additives and artificial substances, may increase inflammation levels, which, in turn, could increase the risk of glaucoma. However, more research is needed to better understand the long-term effects of food additives on human health.

It is also possible that the relationship between UPF consumption and the development of glaucoma could be related to the energy intake of the individual. While the analysis was adjusted to account for energy intake, it is possible that a diet lower in UPF leads to a lower overall energy intake and caloric restriction. Increased UPF consumption has been negatively associated with the nutritional quality of the diet, including increasing the intake of free sugars, total fats, and saturated fats while decreasing fiber, vitamins, and minerals such as protein, zinc, potassium, and magnesium [40]. This could lead to a greater caloric intake [11]. Supporting this notion, Mehta et al. [41] showed that modifying the diet to include more vegetables, fruits, and grains and reducing fat intake increased the risk of developing glaucoma among women, regardless of race/ethnicity or age. Although there is promising research on the effects of caloric restriction in preclinical animal studies, there is currently no information on its effects on patients with glaucoma [42]. However, it is important to note that a retrospective cohort study found that diabetic patients using the hypoglycemic drug metformin had a decreased risk of developing primary open-angle glaucoma. This effect is believed to be similar to the health benefits of caloric restriction and to activate pathways associated with longevity [43].

Another factor to consider is the impact of consuming highly processed foods on nutrient intake. Although the connection between UPFs and health problems is not yet fully understood [8], it is believed to be due to their poor nutritional content, including high levels of added sugars, trans-fat, and sodium, as well as the displacement of healthier, unprocessed or minimally processed foods in the diet [44]. A major concern is the nutrient displacement that occurs when UPFs replace healthier options [45], such as fruits and vegetables [46]. It has been reported that a greater consumption of green leafy vegetables and nitrates is related to a lower risk of primary open-angle glaucoma, mainly in early cases of paracentral visual field loss at diagnosis [47]. Additionally, consuming fewer fruits and vegetables can lead to a reduction in polyphenol intake, which has multiple potential health benefits, including antioxidant and anti-inflammatory properties [32]. Similarly, a lower intake of these healthy foods could lead to reductions in carotenoid intake (i.e., lutein, zeaxanthin) [48]. These carotenoids accumulate in the retina to form macular pigment, with emerging evidence indicating a relationship between macular pigment levels and age-related eye diseases (e.g., glaucoma) [49].

This study was performed in the presence of limitations that must be declared. This is an observational study, so it is not possible to disregard the possibility of residual confounding, as there may be unmeasured confounders. Nevertheless, the multivariate analysis considered many potential confounders (i.e., sex, age, physical activity, tobacco use, omega 3:6 ratio, and energy intake). The cohort consists primarily of university graduates, over 50% of whom are healthcare professionals, which enhances the quality of self-reported data but reduces the representativeness of the sample. The SUN cohort consists mainly of healthy middle-aged college graduates; thus, the prevalence of glaucoma in the sample is low since it increases with age. However, the large sample size provides enough statistical power for the analysis. It is worth mentioning that food consists of multiple chemical components that interact with each other, making it challenging to determine its relationship with disease. This is because nutrients are consumed as part of a dietary pattern rather than individually [50]. Last, the food frequency questionnaire used was not tailored to collect data on the consumption of foods under the NOVA classification of UPF, so the study did not include certain items such as energy bars, energy drinks, health and slimming products, and meat or vegetable nuggets, which could result in an underestimate of UPF consumption. Conversely, this study has several strengths that should be mentioned, such as the use of repeated measures, the long follow-up period, the large sample of participants in the SUN cohort, and the high retention rate (93.0%). In addition, stratified analyses by different covariates were carried out, giving robustness and consistency to the results obtained.

## 5. Conclusions

This prospective cohort study shows that participants with a greater UPF consumption have a higher risk of developing glaucoma in comparison with participants with a lower consumption. Our findings emphasize the relevance of monitoring and limiting the consumption of UPFs (especially those rich in sugar) to prevent glaucoma incidence. Given the increasing trends of UPF consumption in our society, promoting adherence to an unprocessed or minimally processed food pattern should be encouraged. Additionally, implementing measures such as front-of-package labeling, taxation on unhealthy foods, restrictions on advertising, and promoting healthier options, as seen in some countries, can further discourage the consumption of UPFs. We consider that UPFs can be easily identified, so the advice of limiting their intake for promoting glaucoma health in the clinical consultation can be straightforward.

## Figures and Tables

**Table 1:** Classification of foods in the SUN food frequency questionnaire according to the degree of processing (NOVA).

Group	Included Foods
Unprocessed or minimally processed foods	Vegetables, fruit, grains (including white rice and pasta), legumes, milk (whole, semi-skimmed, and nonfat), eggs, meats, poultry, fish and seafood, fermented milk like yogurt, water, natural juice, and coffee.
Processed culinary ingredients	Vegetable oils (olive, sunflower, corn), lard, sugar, chili, butter salt and honey.
Processed foods	Breads (both white and whole), cured traditional ham, bacon, condensed milk, cream, various cheeses, canned and bottled fruit, wine, and beer.
Ultra-processed foods	Potato chips, pizza, preprepared pies, breakfast cereals, margarine, cookies and chocolate cookies, doughnuts, muffins, croissants or other non-handmade pastries, cakes, churros, chocolates and candies, marzipan, nougat, carbonated drinks, artificially sugared beverages, fruit drinks, milkshakes, instant creams and soups, mayonnaise, and alcoholic drinks produced by fermentation followed by distillation such as whisky, gin, and rum. Whereas items such as ice cream, *petit-suisse*, flan, pudding, custard, processed meats (chorizo, salami, mortadella, sausage, hamburger, *morcilla*), ham, spicy sausage/meatballs, croquettes, *pâté*, and *foie-gras*.

**Table 2:** Baseline characteristics of participants by servings of ultra-processed foods consumption. Values are expressed as means (SD), unless otherwise noted. The SUN project.

Variable	UPF (up to 1 Serving/Day)	UPF (>1 to 3 Servings/Day)	UPF (>3 to 4 Servings/Day)	UPF (>4 Servings/Day)	*p*-Value
Participants, n (%) ^a^	1004 (5.2)	8237 (42.8)	4196 (21.8)	5818 (30.2)	
UPF, servings/day	0.7 (0.2)	2.1 (0.5)	3.5 (0.3)	5.4 (1.5)	<0.001
Age, years	47.8 (12.6)	40.7 (12.6)	36.5 (11.6)	34.3 (10.8)	<0.001
Sex, women, n (%)	643 (64.0)	5163 (62.7)	2587 (61.7)	3176 (54.6)	<0.001
University education, years	5.1 (1.6)	5.1 (1.5)	5.0 (1.5)	5.0 (1.5)	0.380
BMI, kg/m^2^	24.0 (3.5)	23.7 (3.6)	23.4 (3.5)	23.4 (3.5)	<0.001
Physical activity, METs-h/week	22.8 (23.5)	21.5 (22.6)	21.3 (21.8)	22.5 (24.2)	0.012
Total energy intake, kcal/day	1687 (542)	2074 (529)	2404 (497)	2777 (515)	<0.001
Carbohydrates intake, % of energy	32.8 (8.5)	35.5 (6.8)	36.7 (6.0)	37.5 (6.0)	0.003
Protein intake, % of energy	20.6 (4.9)	19.0 (3.4)	18.1 (2.8)	17.0 (2.7)	<0.001
Fat intake, % of energy	44.1 (10.2)	43.3 (7.8)	43.3 (6.8)	43.6 (7.0)	<0.001
Adherence to the MedDiet, 0 to 9 score	4.9 (1.7)	4.4 (1.8)	4.1 (1.8)	3.8 (1.7)	<0.001
Omega 3:6 intake ratio	0.3 (0.3)	0.2 (0.1)	0.2 (0.1)	0.1 (0.1)	<0.001
Caffeine intake, mg/day	34.1 (38.3)	38.3 (36.5)	42.1 (36.4)	49.9 (45.0)	<0.001
Ethanol intake, g/day	6.0 (10.6)	6.4 (9.9)	6.5 (9.0)	7.4 (11.2)	<0.001
Smoking, packages-year ^b^	10.3 (13.5)	7.2 (10.8)	5.5 (9.1)	4.8 (8.6)	<0.001
Special diet, yes, n (%)	190 (18.9)	826 (10.0)	293 (7.0)	308 (5.3)	<0.001
Cancer, n (%)	45 (4.5)	258 (3.1)	104 (2.5)	105 (1.8)	<0.001
Hypertension, n (%)	190 (18.9)	1010 (12.3)	376 (9.0)	487 (8.4)	<0.001
Type 2 diabetes, n (%)	52 (5.2)	181 (2.2)	51 (1.2)	76 (1.3)	<0.001
CVD, n (%)	37 (3.7)	151 (1.8)	52 (1.2)	64 (1.1)	<0.001

BMI, body mass index; CVD, cardiovascular disease; METs, metabolic equivalents of task; UPF, ultra-processed food. ^a^ At baseline. ^b^ Numbers of packs (20 cigarettes)/day multiplied by years of smoking.

**Table 3:** Risk of glaucoma (hazard ratio and 95% confidence intervals) according to servings of ultra-processed foods consumption. The SUN Project.

Variable	UPF (up to 1 Serving/Day)	UPF (>1 to 3 Servings/Day)	UPF (>3 to 4 Servings/Day)	UPF (>4 Servings/Day)	For Each Serving/Day Increment	*p* for Trend
Participants, n (%) ^a^	961 (5.0%)	8585 (44.6%)	4292 (22.3%)	5417 (28.1%)	19,255	
Glaucoma cases, n (%)	24 (2.5%)	156 (1.8%)	61 (1.4%)	73 (1.3%)	314	
Persons/year	12,540	107,286	54,899	73,563	24,828	
Model 0, HR (95% CI)	1 (Reference)	1.16 (0.75–1.79)	1.22 (0.76–1.97)	1.36 (0.85–2.17)	1.04 0.97–1.11)	0.172
Model 1, HR (95% CI) ^b^	1 (Reference)	1.28 (0.83–1.97)	1.41 (0.87–2.27)	1.61 (1.00–2.50)	1.07 (1.00–1.14)	0.009
Model 2, HR (95% CI) ^b^	1 (Reference)	1.30 (0.84–2.01)	1.44 (0.89–2.34)	1.65 (1.02–2.67)	1.07 (1.00–1.14)	0.091
Model 3, HR (95% CI) ^b^	1 (Reference)	1.42 (0.90–2.23)	1.62 (0.95–2.74)	1.82 (1.04–3.16)	1.07 (0.98–1.16)	0.004
Model 4, HR (95% CI) ^b^	1 (Reference)	1.43 (0.91–2.26)	1.66 (0.98–2.81)	1.84 (1.06–3.21)	1.07 (0.99–1.16)	0.005

CI, confidence interval; HR, hazard ratio; UPF, ultra-processed food. ^a^ At the end of the follow-up. ^b^ Stratified by decades of age and recruitment period. Model 0, unadjusted; Model 1, adjusted for sex and age; Model 2, adjusted for Model 1 + sociodemographic variables (educational level); Model 3, adjusted for Model 2 + lifestyle and diet variables (leisure time physical activity, total energy intake, adherence to the Mediterranean diet, omega 3:6 ratio, caffeine intake, ethanol intake, cigarettes smoked per year, special diets); Model 4, adjusted for Model 3 + participant’s medical history (body mass index at baseline, cancer at baseline, hypertension at baseline, type 2 diabetes at baseline, cardiovascular disease at baseline).

## Data Availability

The original contributions presented in the study are included in the article/Supplementary Materials, further inquiries can be directed to the corresponding author.

## Supplementary Materials

The following supporting information can be downloaded at: https://www.mdpi.com/article/10.3390/nu16071053/s1, Table S1: Ad-hoc ultra-processed foods classification in the SUN food frequency questionnaire.
