# Supplementary material for: Is Ultra-Processed Food Intake Associated with a Higher Risk of Glaucoma? A Prospective Cohort Study including 19,255 Participants from the SUN Project
Source: Nutrients. 2024 Apr 4;16(7):1053. doi: 10.3390/nu16071053 (PMC11013077; doi:10.3390/nu16071053)
Supplement: Supplementary file 1 [file nutrients-16-01053-s001.zip › nutrients-2900193-supplementary.pdf]

Table S1. *Ad-hoc* ultra-processed foods classification in the SUN food frequency questionnaire.

Group	Included foods
Sausages	Processed meat (chorizo, salami, mortadella, sausage, morcilla), ham, spicy sausage/meatballs, pâté, and foie-gras.
Beverages	Carbonated drinks, artificially sugared beverages, fruit drinks, alcoholic drinks produced by fermentation followed by distillation such as whisky, gin, and rum.
Fried foods	Potato chips, croquettes, and other fried foods.
Sweets	Pre-prepared pies, breakfast cereals, cookies and chocolate cookies, doughnuts, muffins, croissants or other non-handmade pastries, cakes, churros, chocolates and candies, marzipan, and nougat.
Fast food	Pizza, hamburger, instant soups and creams, and mayonnaise.
Dairy products	Ice cream, custard, *petit-suisse*, flan, pudding, milkshakes, and margarine.
